# Assessing and ensuring fidelity of the nationally implemented English NHS diabetes prevention programme: lessons learned for the implementation of large-scale behaviour change programmes

**DOI:** 10.1080/21642850.2022.2077205

**Published:** 2022-05-23

**Authors:** Rhiannon E. Hawkes, Lisa M. Miles, Peter Bower, Sarah Cotterill, David P. French

**Affiliations:** a Manchester Centre of Health Psychology, Division of Psychology and Mental Health, University of Manchester, Manchester, UK; b Division of Population Health, Health Services Research & Primary Care, University of Manchester, Manchester, UK

**Keywords:** Intervention fidelity, diabetes prevention, programme implementation, health behaviour change, implementation science

## Abstract

**Background:**

Health services interventions are typically more effective in randomised controlled trials than in routine healthcare. One explanation for this ‘voltage drop', i.e. reduction in effectiveness, is a reduction in intervention fidelity, i.e. the extent to which a programme is implemented as intended. This article discusses how to optimise intervention fidelity in nationally implemented behaviour change programmes, using as an exemplar the National Health Service Diabetes Prevention Programme (NHS-DPP); a behaviour change intervention for adults in England at increased risk of developing Type 2 diabetes, delivered by four independent provider organisations. We summarise key findings from a thorough fidelity evaluation of the NHS-DPP assessing design (whether programme plans were in accordance with the evidence base), training (of staff to deliver key intervention components), delivery (of key intervention components), receipt (participant understanding of intervention content), and highlight lessons learned for the implementation of other large-scale programmes.

**Results:**

NHS-DPP providers delivered the majority of behaviour change content specified in their programme designs. However, a drift in fidelity was apparent at multiple points: from the evidence base, during programme commissioning, and on to providers’ programme designs. A lack of clear theoretical rationale for the intervention contents was apparent in design, training, and delivery. Our evaluation suggests that many fidelity issues may have been less prevalent if there was a clear underpinning theory from the outset.

**Conclusion:**

We provide recommendations to enhance fidelity of nationally implemented behaviour change programmes. The involvement of a behaviour change specialist in clarifying the theory of change would minimise drift of key intervention content. Further, as loss of fidelity appears notable at the design stage, this should be given particular attention. Based on these recommendations, we describe examples of how we have worked with commissioners of the NHS-DPP to enhance fidelity of the next roll-out of the programme.

## Background

Intervention fidelity is defined as the extent to which an intervention is implemented as intended (Bellg et al., 2004). Without a fidelity assessment, it cannot be ascertained whether intervention (in)effectiveness is due to intrinsic intervention features or factors added or omitted during implementation (Bellg et al., 2004). Large-scale programmes sometimes commission several different providers (private, state or third sector) to deliver the programme on their behalf, following central guidance, with some room for interpretation. Thus, assessing fidelity of large-scale programmes is particularly important due to the involvement of different people at each stage of the programme, increasing the risk of a drift in fidelity through each stage of implementation. Further, tensions between adaptations to different populations and adapting pre-existing programmes to fit specifications present further challenges to fidelity.

The present article considers drift in intervention fidelity in the roll-out of the National Health Service (NHS) England Diabetes Prevention Programme. NHS England commissioned the NHS Diabetes Prevention Programme (NHS-DPP), which is a nationally implemented behaviour change programme for adults in England at high risk of developing Type 2 diabetes (T2DM), following a review of the international evidence for diabetes prevention programmes (Ashra et al., 2015), and a pilot of the programme (Penn et al., 2018). The development of the NHS-DPP drew upon widespread experience of such programmes internationally, with previous diabetes prevention trials from multiple countries (e.g. Knowler et al., 2002; Tuomilehto et al., 2001) suggesting lifestyle programmes to be effective in promoting behavioural change and reducing the incidence of T2DM. These results have translated into routine practice (Aziz, Absetz, Oldroyd, Pronk, & Oldenburg, 2015; Dunkley et al., 2014), albeit with smaller effect sizes. Despite these smaller effects indicating a drift in fidelity, intervention fidelity has not been systematically considered in any of these countries.

The NHS-DPP was delivered by four independent provider organisations between 2016 and 2019, who worked independently to deliver the programme across England (Valabhji et al., 2020). This commissioning approach allows the programme to be delivered at scale and with efficiency, and permits local health services a choice of provider approaches in order to adapt to local context. NHS England stipulated intervention content of the programme within a published Service Specification (NHS England, 2016) based on current evidence (Ashra et al., 2015, National Institute for Health and Care Excellence (NICE), 2012), which the four providers had to adhere to. This specified a minimum of 13 face-to-face group sessions of no more than 15–20 adults with non-diabetic hyperglycaemia, across nine months. A description of the NHS-DPP service and referral process are detailed elsewhere (Hawkes, Cameron, Cotterill, Bower, & French, 2020; Howells, Bower, Burch, Cotterill, & Sanders, 2021). The use of behaviour change techniques (BCTs) such as setting behavioural goals were intended as the ‘active ingredients’ of interventions designed to change behaviour (Michie et al., 2013). The Service Specification emphasised the importance of BCTs to self-regulate behaviour (e.g. action planning, self-monitoring) as core components of the intervention relevant to T2DM prevention (NHS England, 2016).

Multiple aspects of fidelity can be assessed, including design (i.e. whether programme plans were in accordance with the evidence base), training (of staff to deliver intervention components), delivery (of intervention components), and receipt (i.e. participant understanding of intervention content) (Bellg et al., 2004). Most fidelity research concerns interventions developed by those delivering the evaluation, and previous assessments of diabetes prevention programmes have presented a superficial fidelity assessment, for example, defining fidelity only as whether the intervention was based on a standard curriculum (Aziz et al., 2015). By contrast, there are very few independent fidelity assessments of behaviour change interventions applying robust fidelity frameworks, and there are even fewer that assess large-scale programmes (Lorencatto, West, Christopherson, & Michie, 2013) and none that involved multiple providers.

**Figure 1.**
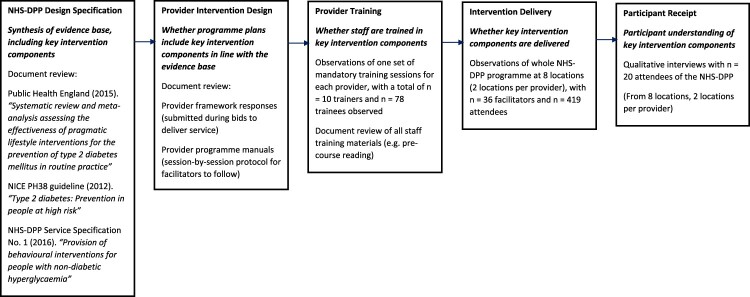
Schematic showing aspects of intervention fidelity assessed in the NHS-DPP.

This article aims to highlight lessons for implementation of large-scale programmes using our fidelity evaluation of the NHS-DPP as an exemplar. Figure 1 illustrates a schematic of each fidelity domain assessed in the NHS-DPP. Consideration of multiple domains allows the drift between intended and actual delivery to be investigated, highlighting the dynamic nature of fidelity over time. Previous fidelity assessments have mainly focused on fidelity of delivery (McGee, Lorencatto, Matvienko-Sikar, & Toomey, 2018). However, if other domains of fidelity are not accounted for, accurate conclusions cannot be drawn about what happened in an intervention and why (Toomey et al., 2020). Thus, our evaluation provides a more comprehensive and systematic assessment of fidelity. We begin by briefly summarising the findings from our fidelity evaluation of the NHS-DPP. Based on these findings, we suggest recommendations for promotion of fidelity for large-scale programme implementation, which we expect would be of particular interest to commissioners and provider organisations.

## Key findings

### Programme design

Assessing fidelity of design of large-scale programmes is different from the more usual assessments of fidelity within research teams due to the involvement of different stakeholders throughout the intervention design. Specifically, these stakeholders include those synthesising the evidence base, commissioners producing a specification, independent providers designing or adapting their programmes based on this specification, and local NHS organisations who choose the provider. Thus, the risk of a drift in fidelity from the evidence base to providers’ programme plans is higher in large-scale programme implementation.

**Figure 2.**
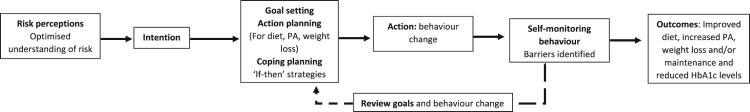
Simplified logic model illustrating how the NHS-DPP is expected to work in achieving health outcomes.

If the theory in behaviour change programmes is poorly chosen, not articulated, or not translated into practice, it will not provide a clear rationale for what should be included in the programme. A logic model is one way to represent a theory of how and why the intervention is expected to work, illustrating anticipated causal pathways between the techniques and desired outcomes (Skivington et al., 2021). Neither the commissioners nor the providers developed an explicit logic model for the NHS-DPP. Thus, we developed a logic model detailing the specific BCT content of the NHS-DPP based on the specification documents (summarising the up-to-date evidence base) underpinning the programme (Ashra et al., 2015; NHS England, 2016, National Institute for Health and Care Excellence (NICE), 2012). Our logic model proposed that information on the risk of T2DM was provided to patients at risk in order to promote formation of behaviour change intentions, followed by a self-regulatory cycle including goal setting and monitoring behaviour, to produce desired behavioural changes and reduction in T2DM risk (Hawkes, Miles, & French, 2021) (see Figure 2). This logic model included a more detailed description of the BCT content and expected mechanisms of action to achieve the desired outcomes in the NHS-DPP, compared to the one developed by Penn et al. (2018) during evaluation of the pilot NHS-DPP in early stages of implementation.

We extracted information on underpinning theory in providers’ programmes using Michie and Prestwich’s Theory Coding Scheme (Michie & Prestwich, 2010). We extracted information on programme format and BCTs using the Template for Intervention Description and Replication framework (Hoffmann et al., 2014) and the BCT Taxonomy v1 (Michie et al., 2013) to determine whether the planned content and BCTs in each of the four providers’ programmes had been designed with fidelity to the NHS-DPP programme specification which was based on the evidence base (Hawkes, Cameron, Bower, & French, 2020). The NHS-DPP programme specification documents (NHS England, 2016, National Institute for Health and Care Excellence (NICE), 2012) were compared with each providers’ programme plans regarding programme format and BCTs.

All four providers had good fidelity of design to the programme format, including planned programme duration, frequency of sessions, and planned group sizes. However, we found variation and a drift in fidelity of BCTs in each providers’ programme plans; providers planned to deliver 74% of specified BCTs (Hawkes, Cameron, Bower, et al., 2020). Some of the key BCTs that were missing from some providers’ programme designs included ‘review outcome goals’, ‘pros and cons’, ‘credible source’, and ‘graded tasks’. Overall, justification for providers’ planned BCTs was not clear, varied between providers, and were often not related to the logic model based on the design specification underpinning the programme (Hawkes, Miles, et al., 2021). Given that some drift in programme delivery was expected, this lack of fidelity to the NHS-DPP programme specification at the intervention design stage is significant. This highlights the complexity of transferring detailed research-based behavioural interventions into consistent plans across multiple providers, and suggests the need for commissioners to review the granular detail of programme plans to ensure they are in line with the evidence base.

### Staff training

A fidelity assessment of staff training of intervention content in large-scale programmes is important because if lack of fidelity in the delivery of the programme is detected, it needs to be clear whether this is due to ineffective training or other contextual factors in programme implementation. Two members of the research team who underwent training in BCT coding and the BCT Taxonomy (BCTTv1 Online Training, n.d.) observed one set of mandatory training courses for each provider. We coded BCTs that staff were trained in during those sessions, including the coding of associated training materials, to evaluate whether the four NHS-DPP providers trained their staff to deliver BCT content with fidelity to their programme plans. We also assessed how thoroughly staff had been trained in BCTs (e.g. whether staff were informed about, directed, instructed, demonstrated, practiced, or modelled BCT delivery) (Hawkes, Cameron, Miles, & French, 2021).

Overall, the training courses for staff covered between 46% and 85% of BCTs in the provider programme plans, thus staff were not trained in a number of planned BCTs. One explanation for this could be the lack of explicit underpinning theory or a logic model from the outset, i.e. a lack of clear rationale for intervention contents in the programme. The most commonly used method of training was instructing how to deliver a BCT, rather than practicing or modelling BCT delivery (Hawkes, Cameron, et al., 2021). We concluded that providers may need to incorporate more comprehensive BCT training to ensure that staff are given the opportunity to practice BCT delivery during their training courses. If staff were better trained in how to deliver the more complex self-regulatory BCTs in a group setting (see Figure 2), this would increase their understanding of how those BCTs work in changing behaviour.

### Programme delivery

An assessment of delivery fidelity is important because if providers are not delivering the BCTs highlighted by the evidence base, it would make the programme less effective and would be difficult to establish reasons for programme (in)effectiveness (Bellg et al., 2004). We assessed fidelity of delivery of the NHS-DPP to (a) each of the four providers’ programme plans and (b) the programme specification (which was based on the evidence base) from observations of the whole NHS-DPP programme in eight locations across England (i.e. two locations per provider) (French, Hawkes, Bower, & Cameron, 2021). Two members of the research team conducted the observations, and each underwent training in the BCT taxonomy (BCTTv1 Online Training, n.d.) prior to data collection.

We found that between 47% and 68% of BCTs included in the programme specification were delivered. Conversely, between 70% and 89% of BCTs included in providers’ programme plans were delivered. There is no clear consensus for what constitutes ‘good’ fidelity, however, there is a general view that > 80% demonstrates ‘high’ fidelity and < 50% demonstrates ‘low’ fidelity (Borrelli, 2011; Lorencatto et al., 2013). Thus, fidelity to programme specification was low to moderate but fidelity to programme plans was generally high. There was extensive delivery of those BCTs which did not require recipients to enact them, notably providing information about health consequences. There was under delivery of BCTs involving self-regulation of behaviours (e.g. problem solving, reviewing goals) (French et al., 2021), which were emphasised as important by the specification documents underpinned by the evidence base (see Figure 2).

Further, an in-depth assessment of the quality of goal-setting delivery in the NHS-DPP found that this technique was not delivered in line with what the goal setting literature suggests is the most effective for changing health behaviours (Hawkes, Warren, Cameron, & French, 2021). For example, although providers generally encouraged setting specific goals, service users were not encouraged to make a public commitment to their behaviour change and the reviewing of goals was rarely specified (Hawkes, Warren, et al., 2021). This may highlight the need for programme developers and delivery staff to be more thoroughly trained in effective delivery of self-regulatory BCTs, for example by demonstrating effective BCT delivery and allowing staff to practice delivery of BCTs during training sessions (Hawkes, Cameron, et al., 2021).

Our results suggested that there was a gap between what the evidence base indicated is most effective and programme delivery, largely because of failures to translate the evidence base into the providers’ programme plans (French et al., 2021). This illustrates the higher likelihood of a drift in fidelity of delivery of BCTs when there are experts, commissioners, and providers involved at different stages of programme implementation. More consistent use of a logic model would produce greater clarity in reasons for intervention content, which may prevent this drift in fidelity.

### Participant receipt

Despite the importance of self-regulatory BCTs in behaviour change programmes, little is known about how participants understand these BCTs, and qualitative evaluation of receipt is rare (Hankonen, 2021). Even the most rigorously designed interventions delivered with perfect fidelity will be ineffective at achieving desired behaviour changes if the intervention content is not understood by recipients (Hankonen, 2021).

We conducted 20 telephone interviews with participants who attended one of eight NHS-DPP programmes that the research team observed (Miles, Hawkes, & French, 2021). Participants were asked about their understanding of self-regulatory BCTs, because those were regarded in the specification as essential for behaviour change (see Figure 2). When asked about their understanding of a BCT, user-friendly examples were used to explain what was meant by that BCT and/or prompts were used that referred to relevant delivery material, to help account for differing levels of health literacy across participants. The topic guide was used flexibly in interviews to allow discussion of enactment of BCTs when participants spontaneously shared relevant experiences.

Our research found that participants generally understood the BCTs ‘self-monitoring of behaviours’ and ‘feedback on outcomes’, but the extent to which participants understood ‘goal setting’ and ‘problem solving’ varied. There was a limited recall and understanding of ‘action planning’ across most participants (Miles et al., 2021). Some of these findings were consistent with our observations of programme delivery across providers. For example, the BCTs ‘self-monitoring of behaviours’ and ‘feedback on outcomes’ were delivered frequently, and participants described an understanding of these BCTs. There was wide variation in the frequency of delivery of ‘goal setting’ and ‘problem solving’ across providers, which may explain the variation in participant understanding of these BCTs, and ‘action planning’ tended to be delivered infrequently, which may account for the limited recall of this BCT across participants (Miles et al., 2021).

Our findings on lack of understanding of some self-regulatory BCTs could be due to the under-delivery of these BCTs (French et al., 2021; Hawkes, Warren, et al., 2021), or the lack of comprehensive staff training of some of these techniques (Hawkes, Cameron, et al., 2021), or both. The large variation in understanding of BCTs between participants (especially goal setting and problem solving) could be attributed to variation in delivery and training across providers, though it is possible that some BCTs, such as self-monitoring of behaviours, may be intrinsically easier to understand and less dependent on frequency of delivery. The lack of understanding of these self-regulatory BCTs would influence the extent to which programmes produce long-term effects in behaviour change.

## Discussion

The present programme of research examining fidelity of the NHS-DPP is the most comprehensive evaluation of a nationally implemented programme to date, thus it can provide lessons learned for other large-scale programmes internationally. Early outcomes from the NHS-DPP suggest that the programme appears effective (Valabhji et al., 2020), but improvement of future programme implementation and adherence to the evidence base could increase the likelihood of achieving sustained outcomes (Dunkley et al., 2014). We suggest recommendations for commissioners and providers to increase fidelity of behaviour change content in other large-scale programmes (see Table 1). We also highlight some policy changes that have happened in the NHS-DPP in response to these recommendations (Table 1).

**Table 1.** Recommendations to enhance fidelity of nationally implemented programmes.

Domain of fidelity	Author recommendations	Advantages of employing these methods	Enhancements made to the NHS-DPP
**Design**	There should be an explicit theory informing the programme design to provide a clear description of how the intervention expects to produce changes in behaviour. Large-scale programmes could benefit from commissioners providing a logic model from the outset to guide providers to adapt the logic model for their own programmes. Alternatively, it could be a requirement of providers to specify a logic model as part of the commissioning process.	Providers have a clear rationale for the BCTs in the evidence base. Fewer issues with fidelity expected if explicit theory is specified from the outset. Facilitates testing of exactly which behaviour change programmes work in changing health behaviours and why.	NHS England now require providers to include a logic model or table in their service bids detailing which BCTs they have included in their programme designs and how they expect these techniques to achieve the desired programme outcomes.
	Commissioners need to be more explicit about what they expect to be included in the programme, i.e. be clear about criteria providers will be evaluated against. Commissioners should ensure a robust quality assurance process, starting from the bidding process, to help detect gaps in the intervention design plans. Commissioners should ensure the evidence base translates into the contents of intervention plans, i.e. that providers are planning to use intervention techniques for which there is the strongest evidence.	More justified planning during the bidding process could save time and money when trying to achieve the desired outcomes as programmes are in line with the evidence base regarding effectiveness from the outset.	Members of present research team were involved in revising the wording of behaviour change content requirements in the NHS-DPP Service Specification, based on the findings from this programme of research. NHS England now require providers to explicitly describe how they will support service users with self-regulatory techniques, including support with setting, monitoring and reviewing of goals. Providers have to explain how their intervention has been developed with behaviour change specialists. Members of the present research team who have expertise in behaviour change were involved in evaluating provider bids during the commissioning of the third wave roll-out of the NHS-DPP to ensure that those providers bidding to deliver the service included behaviour change content that was in line with the current evidence base.
	Once best providers have been commissioned, they could be further supported to ensure planned programmes meet the requirements of the evidence base. Specialists in behavioural science should be involved to ensure the core components are assessed before roll-out. If the above is not possible, contract management arrangements should ensure programme fidelity and use of behavioural science is monitored and part of continuous improvement activity.	Interventions would be grounded in theory and in line with the current evidence base from the outset. Programmes less likely to omit the most effective intervention techniques for their populations/contexts.	NHS England now further emphasise for providers to set out during the bidding process how they would ensure fidelity and continuous improvement.
**Training**	Commissioners should be clear about the minimum level qualifications and experience of staff that providers should employ to deliver the programme. Commissioners could follow-up with providers to ensure their employed staff have the minimum level of qualifications required.	Ensures that providers are employing appropriate staff to deliver the more complex behaviour change content of the programme.	NHS England now require providers to state in their service bids what behaviour change training staff will receive before delivering the service.
	Providers should ensure that staff are trained in all important intervention components present in their intervention design. A behaviour change specialist could be involved in developing and delivering staff training, which should focus on training of BCTs. Training of BCTs should be appropriate to the format of the session (e.g. group delivery, telephone calls, etc.) and population (e.g. tailoring of techniques such as appropriate dietary examples for population).	BCTs are more likely to be delivered if staff have received thorough training in these components of the intervention. Staff would receive in-depth training into the underpinning theory of techniques to change behaviours. Appropriate training should result in more effective delivery of those techniques, which should improve fidelity of participant receipt.	NHS England require evidence from providers that relevant health professionals or specialists are involved in development of the intervention, including in the training of staff.
	Commissioners could advise providers (with guidance from behavioural science expertise) on a minimum amount of training days/time that should be spent on training delivery staff in specific behaviour change content, and advise on the content and scope of behaviour change training. Trainee staff should receive comprehensive training of BCTs. E.g. trainers should demonstrate how to deliver BCTs and trainee staff should practice intervention content delivery before delivering in routine practice.	Staff would have a more comprehensive understanding of what the BCTs are, why they are important, and how to deliver them in routine practice. Could be especially useful for the training of more complex behaviour change components such as self-regulatory techniques.	NHS England now require providers to state in their service bids how their staff training focuses on behaviour change technique delivery, group management, communication and rapport, and how the training allows front-line staff the opportunity to practice using these skills and techniques.
**Delivery**	Providers should ensure delivery staff receive continued monitoring and feedback from senior members of staff and experts in behaviour change to ensure intervention techniques are delivered optimally in routine practice.	Certifies that staff are delivering the same high standard of BCTs, regardless of their background and previous experience. This will increase competence and confidence of delivering complex behaviour change interventions, including self-regulatory techniques. This would help prevent a drift in delivery fidelity over time. If the quality of delivery of BCTs is improved, this may improve participant receipt and subsequent programme outcomes.	NHS England require providers to state their approach to external quality assurance to monitor and assure fidelity of delivery and the ongoing quality and consistency of delivery of services.
**Receipt**	Researchers should investigate whether particular BCTs in the intervention are understood by participants and which BCTs are more effective for particular populations or intervention formats or settings.	This would improve the evidence base for future interventions and help improve outcomes if implemented.	

### Recommendations for the promotion of fidelity for large-scale programme implementation

There should be an explicit theoretical underpinning of the programme to ensure a clear rationale for inclusion of intervention techniques. A logic model, for example, is one way that multiple theories and evidence sources can be represented diagrammatically. Our analysis of the NHS-DPP identified that many fidelity issues may have been less prevalent if there was a clear underpinning theory from the outset. The inclusion of a logic model during programme design would identify the important BCTs to be translated into programme plans, which may prevent a drift in fidelity at the training and delivery stages of implementation. This recommendation has now been incorporated into the NHS England specification documents for the third wave of commissioning of the NHS-DPP; as a result of the current research, NHS England now require providers to produce a logic model to describe justification for the key intervention components of their programmes, and require providers to explicitly describe how they expect their planned BCTs to work and why.

During the bidding process, commissioners should be clear on the criteria they use to evaluate providers (e.g. clarity of underpinning theory used to select BCTs in intervention design). Robust quality assurance during commissioning would ensure the evidence base is clearly translated into the contents of programme plans. This would reduce the likelihood of a dilution in fidelity in BCTs from the evidence review to commissioning through programme design. Involving a behaviour change specialist during commissioning would ensure gaps in intervention design are detected early on. The research team have since worked with the commissioners of the NHS-DPP for the third wave roll-out of the programme to ensure the BCTs in the evidence base are clearly translated into the NHS-DPP programme specification and the required behaviour change content of the programme is clearly articulated. For example, NHS England now require providers to explicitly describe in their bids how they will support service users with self-regulatory techniques, including support with setting, monitoring and reviewing of goals. Providers who are commissioned to deliver the programme could be further supported to refine their programmes in line with the evidence base before programme delivery; this may be particularly important for providers who have adapted pre-existing programmes for the current specification.

The involvement of a behaviour change specialist at all stages of programme implementation is important. Specialists could deliver the staff training to provide an in-depth understanding on the psychological mechanisms of BCTs, and allowing staff to practice BCT delivery could increase competence (Hawkes, Cameron, et al., 2021). This would be particularly advantageous for the more complex self-regulatory techniques underpinned by the evidence base (e.g. goal setting, problem solving). The training should also be appropriate to the session format (e.g. group delivery) and target population (e.g. tailoring techniques to culture, age, ethnicity, etc.). Thus, commissioners should be explicit about the minimum level of qualifications and training required for staff to deliver complex behaviour change content, and ensure providers allocate enough time for the training of such techniques. In response to these recommendations, during commissioning of the third wave of the programme NHS England required providers to explicitly state in their programme bids how staff would be trained in intervention content, specifying that providers should train staff thoroughly in behaviour change content and group delivery skills and allow enough time for front-line staff to practice intervention content before delivering in the field.

Future work of this nature should also consider changes to fidelity over time, once the programme is implemented in the field. Thus, providers should ensure delivery staff receive continued monitoring and feedback from experts in behaviour change so that BCTs are delivered effectively, which also has the potential to increase participant engagement and adherence with the programme. This highlights the conflict between rapid roll-out of large-scale interventions and prioritising implementation fidelity; without thorough training in these techniques and ongoing audit and feedback, it could result in sub-optimal delivery and limited participant understanding of intervention content, impacting on programme outcomes.

### Strengths and limitations

This programme of research is the first independent evaluation of the fidelity of a nationally implemented diabetes prevention programme in the world. We assessed each domain of fidelity, building on the National Institute of Health’s Behaviour Change Consortium guidance (Bellg et al., 2004), to provide a comprehensive understanding of the behaviour change content included in the NHS-DPP. We have also demonstrated how recommendations arising from this research have been discussed and implemented with stakeholders to improve fidelity of behaviour change content in future roll-outs of the NHS-DPP. However, whilst we hope that these methods will be useful as models for research teams to apply to evaluations of other large-scale programmes with multiple providers, there are limitations to acknowledge.

First, we obtained all relevant documentation from all providers to fully assess the behaviour change content in the design, training, and delivery of the NHS-DPP. However, a challenge of examining fidelity of a programme where the research team were independent of the teams that developed the intervention is that the research team could not ascertain what exactly providers intended to be the ‘active ingredients’ of their interventions, as these were not clearly spelt out. Rather, standardised coding frameworks (e.g. Michie et al., 2013; Michie & Prestwich, 2010) were used to interpret this. Second, providers were considered to demonstrate fidelity when a BCT stated in the full programme specification was present in providers’ intervention design and delivery. With the sole exception of goal setting, we did not assess how well the BCT was delivered. This pragmatic decision was taken, although there is no compelling evidence that use of a technique once is sufficient, and neither is it clear that BCTs were delivered to a standard required to be effective.

A necessary limitation was that we made decisions regarding how much of the NHS-DPP to attempt to capture. With four providers delivering the programme, it would require a large degree of observation of delivery or training to be entirely sure that our fidelity assessment was a thoroughly reliable assessment. For example, the present programme of work observed one complete set of mandatory training courses across providers (Hawkes, Cameron, et al., 2021) and complete delivery of the nine-month NHS-DPP at eight sites involving 35 facilitators observed at 111 sessions (French et al., 2021). Such observation cannot be truly representative of an entire programme, however, we aimed to ensure that the research captured what was delivered in complete courses and observed in diverse geographical locations across England, within the constraints of time and resource. Finally, the majority of service users interviewed about their understanding of the behaviour change content (Miles et al., 2021) had completed all or most sessions of the NHS-DPP and were interviewed after the nine-month programme had finished; participants who had completed less of the programme may have reported differences in their understanding of BCT content and it is possible that participants had some difficulty recalling the detail of the programme at the time of the interview.

### Conclusions

This article identifies lessons learned from a unique fidelity assessment of a nationally implemented behaviour change programme. We suggest recommendations for future implementation of large-scale programmes in which there is a higher risk of a drift in fidelity to ensure interventions are in line with the evidence base regarding effectiveness, and describe examples of policy changes that have happened in the NHS-DPP as a result of this programme of research. These recommendations would incur minor costs, but it is anticipated that they would yield reasonable sized benefits in effectiveness if implemented, through reducing intervention drift.

## Abbreviations

**Abbreviations:** BCT: Behaviour Change Technique; HbA1c: haemoglobin A1C; NHS-DPP: National Health Service Diabetes Prevention Programme; T2DM: Type 2 diabetes
